# P-1790. Phylogenetic Analysis, Mutation Characterization, and Comparison with Vaccine Reference Strains of Influenza HA Protein Sequences During the 2021/2022 and 2024 Seasons in Southwest Guatemala

**DOI:** 10.1093/ofid/ofaf695.1959

**Published:** 2026-01-11

**Authors:** Julio del Cid-Villatoro, Daniel Vásquez, Claudia Paiz, Kareen Arias, Neudy C Rojop, Claire Bradley, Diva M Barrientos, Molly Lamb, Beatriz Lopez, Emily Zielinski-Gutierrez, Daniel Carreon, Ashley Fowlkes, Chelsea Iwamoto, Daniel Olson

**Affiliations:** Fundación para la Salud Integral de los Guatemaltecos, Retalhuleu, Retalhuleu, Guatemala; Fundación para la Salud Integral de los Guatemaltecos, Retalhuleu, Retalhuleu, Guatemala; Fundacion Para La Salud Integral de los Guatemaltecos, Los Encuentros, Retalhuleu, Guatemala; Fundacion Para La Salud Integral de los guatemaltecos, Los Encuentros, Retalhuleu, Guatemala; Fundacion Para La Salud Integral de los Guatemaltecos, Los Encuentros, Retalhuleu, Guatemala; Fundación para la Salud Integral de los Guatemaltecos, Retalhuleu, Retalhuleu, Guatemala; Fundación para la Salud Integral de los Guatemaltecos, Retalhuleu, Retalhuleu, Guatemala; Colorado School of Public Health, Aurora, Colorado; Centers for Disease Control and Prevention Central America, Guatemala, Quetzaltenango, Guatemala; Centers for Disease Control and Prevention Central America, Guatemala, Quetzaltenango, Guatemala; Centers for Disease Control and Prevention, Atlanta, Georgia; Centers for Disease Control and Prevention, Atlanta, Georgia; Center for Disease Control and Prevention, Atlanta, Georgia; CU School of Medicine, Denver, Colorado

## Abstract

**Background:**

Background

Influenza virus surface protein hemagglutinin (HA) is the primary target of antibodies that protect against influenza virus infection and disease. The viral composition of the annual influenza vaccines may differ for northern (NH) and southern (SH) hemisphere seasons. Vaccine formulation for Guatemala has fluctuated between NH and SH; some years both are used.

**Figure:**
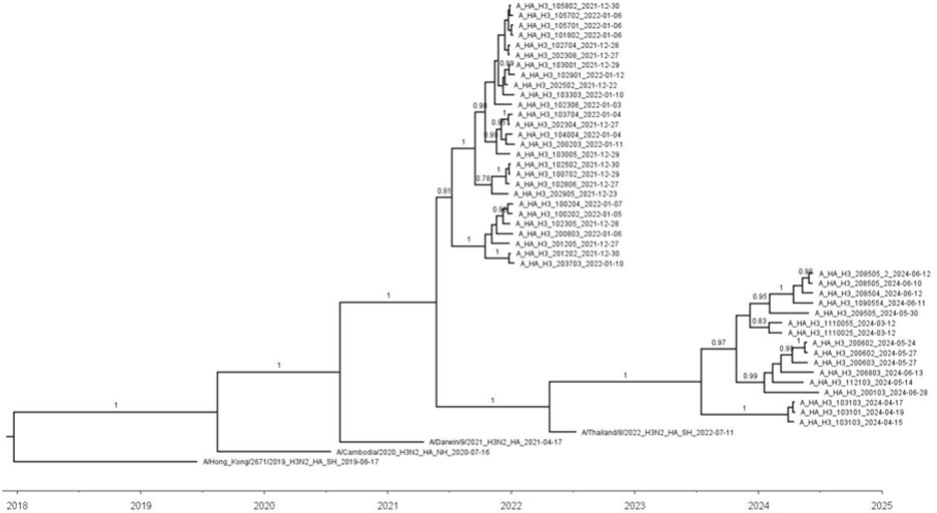
Phylogenetic tree of influenza A H3N2 HA sequences collected in southwest Guatemala from in the 2021/2022 and 2024 seasons Influenza A H3 sequences were aligned along northern and southern hemisphere reference vaccine strains for the respective season. Southern hemisphere reference strain (A/Thailand/8/2022) showed the closest relationship to circulating strains.

**Figure:**
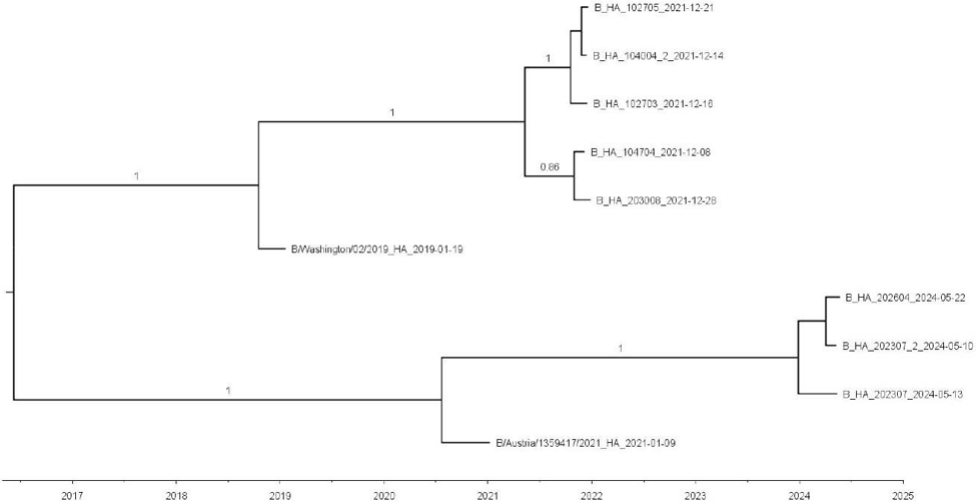
Phylogenetic tree of influenza B HA sequences collected in southwest Guatemala from in the 2021/2022 and 2024 seasons Both reference strains matched the circulating strains' clade for the respective season.

**Methods:**

Methods

We used the MinION (Oxford Nanopore Technologies) to sequence influenza A(H3N2) (n=60) and influenza B (n=9) virus samples collected in southwest Guatemala from December 2021–January 2022 and January–June 2024. HA protein sequence alignment with vaccine strains was conducted. Maximum likelihood trees were generated to estimate evolutionary distance between circulating and vaccine strains and were compared using Wilcoxon sign rank test. Mutation characterization was performed using FluSurver.

**Results:**

Results

For 2021/2022 A(H3N2) samples, we found a mean evolutionary distance of 9.7 amino acid (AA) substitutions/100

sites compared to the SH vaccine strain and 5.9 to NH (SH to NH distance comparison p < 0.0001). None of the vaccine strains

(3C.2a1b.1b/3C.2a1b.2a.1a) shared clades with circulating viruses (3C.2a1b.2a.2a.3). For the 2024 A(H3N2) samples, analysis revealed a mean distance of 2.3 compared to SH and 5.2 to NH vaccine strains (SH to NH distance comparison p < 0.001). Sequences from this period had a shared clade (3C.2a1b.2a.2a.3a.1) with the SH vaccine strain. Mutation characterization identified one of the 2024 samples with a mutation that removes a potential glycosylation site (T151A). Influenza B virus sequences belonged to the same clade as the vaccine viruses (V1A.3 for 2021/2022 and V1A.3a.2 for 2024).

**Conclusion:**

Conclusion

The 2021/2022 circulating sequences in the region showed more AA similarity with the NH A(H3N2) compared with the SH vaccine virus sequence. In contrast, the 2024 circulating A(H3N2) strains showed more AA similarity and shared the same clade with the SH vaccine virus sequence. Our data may inform a proposed Guatemala-wide analysis to evaluate if location-specific NH/SH formulation recommendations could result in a better match between vaccine viruses and circulating strains.

**Disclosures:**

Molly Lamb, PhD, Merck: Grant/Research Support Daniel Olson, MD, Fundacion para la Salud Integral de los Guatemaltecos: Board Member|Merck: Grant/Research Support|Roche Diagnostics: Grant/Research Support

